# Developing Organizational Competences for Conflict Management: The Use of the Prisoner's Dilemma in Higher Education

**DOI:** 10.3389/fpsyg.2018.00376

**Published:** 2018-03-21

**Authors:** Andreina Bruno, Giuseppina Dell'Aversana, Gloria Guidetti

**Affiliations:** ^1^Department of Education Sciences, University of Genova, Genoa, Italy; ^2^Department of Psychology, University of Torino, Turin, Italy

**Keywords:** conflict, higher education, prisoner's dilemma, game-theory, psychoanalytic approach

## Abstract

Interpersonal relationship require sophisticated competences of cohabitation. However, the availability of training tools to develop conflict management skills is limited and problematic. The prisoner's dilemma game (PDG), the most widely known example of game theory, a nonzero-sum game, has been used, in higher education, to provide students with an opportunity of active learning and for understanding counterintuitive concepts. It creates a condition of emotive, moral and decisional conflict in and between agents. This paper presents a case-study in higher education in which PDG was proposed to enhance organizational competences for conflict management, according to the psychoanalytic approach to organizational studies. The study aims to explore: (1) the significant characteristics of PDG used in an affective-emotional key in higher education; (2) the learning outcomes that PDG enables to activate in the participants in relation to the development of organizational skills for conflict management. Twenty students' reflective journals were analyzed using thematic analysis. Findings indicated that PDG is perceived as a useful device in students' learning experience, which is appreciated in relation to its concreteness, intensity and debriefing phase. Learning outcomes allow new meanings about conflict, by emphasizing its defensive, automatic and interpersonal dimension. This paper contributes to the understanding of PDG as a tool to develop competences in dealing with the challenges of conflict management, since it seems to favor the overcoming of the individualistic stereotype in conflict representation by highlighting the interdependence of social interaction.

## Introduction

In the organizational studies debate, conflict has been intended as a guilt, as a yoke, or as a resource. The different conceptions of conflict are anchored with the different possible conceptions of organization (Maggi, 1995). Depending on how it is intended, organizational competences for conflict management require to space by the logic of suppression, that of avoidance, up to the logic of management that sees conflict as a resource of the social system.

In the classical perspective of organization, the conflict is understood as a sign of social malfunction, human incorrect execution and individual guilt, as it is the outcome of a bad joint between the individual and the predefined system. This organizational perspective represents the conflict as not tolerable, and expected to be suppressed: in this way, it favors the reference to the individualistic stereotype in describing conflict management competences (Bruno, 2018).

In more recent views, conflict has been intended as a constituent part of the social system, conceptualized as a process in motion. Conflict represents the irreducible tension between the different organizational dimensions. This perspective emphasizes the need for organizational competences in staying in motion between differences (Augier and March, 2001) and, in particular, in managing conflicts (Bradley and Monda-Amaya, 2005). In this conception, conflict is a resource, because it allows to solicit the imagination, move the status quo, and activate innovation within the organization.

The assumption is that in social functioning contradictory logics are coexistent: there is conflict between individual and social desires, between singular desires of one's own and others, between rational and unwitting desires, etc. In particular, the psychoanalytic approach to the study of organizations (Obholzer and Roberts, 1994; Kaneklin, 2009) brought a significant contribution to the understanding of the irrational dimension of social phenomena and their complexity. It allowed to deepen and go beyond the traditional antithesis between cooperative and competitive logics. Every social situation is governed by unconscious collusive modalities of emotionally symbolizing the context by those who belong to that context, collusive modes that, just as perceptive categories serve to perceptively categorize reality, they serve to categorize the reality emotionally. The absolute primitive emotional category is friend-enemy (Carli, 2006). Symbolizing others as an enemy is functional to a simplistic management of conflict in which the dimension of encounter with the extraneous is bypassed to a predictable ritual of clash or escape from the other. Furthermore, the symbolization of others as an enemy is functional to the so-called “zero-sum game” relationship, where there is expected to be a winner and a loser, a situation that is socially reversible (e.g., a game of chess: in that situation the social game falters in a winner who is such because there is a loser; but in the next game the result can be changed). However, relationships between residents and migrants, between women and men, between workers and people looking for work, between new and old generations within organizations, if structured as zero-sum games, see reduced the possibility of meeting with others, to the typical friend-enemy relationship (Viotti et al., 2015). The tragedy of many social situations and the resulting fragmentation stems from the improper transposition of this collusive model into contexts that would require more sophisticated skills of cohabitation (Gozzoli, 2016). The risk of social fragmentation in contemporary societies, when human beings come to see themselves as individuals less connected to other human beings (Taylor, 1991), requires to improve organizational competences for conflict management. Unfortunately, the availability of training tools to develop conflict management skills is limited and problematic (Olson-Buchanan et al., 1998).

## The prisoner's dilemma

In higher education, an active learning practice that can be used to develop competences in the management of conflicts is the prisoner's dilemma game (PDG). PDG is the most widely known example of game theory, modeling a situation that offers different rewards for multi-agent interactions in competitive and cooperative situations, and where the outcome is determined by both agents' choices. It is a nonzero-sum game: whatever benefit accrues to one agent does not necessarily imply a similar penalty imposed on the other one. The game creates a condition of emotive, moral and decisional conflict in and between agents.

There are some disciplines, where Game Theory has been extensively used, like Artificial Intelligence, Economics, Biology, Mathematics and Social Sciences (Burguillo, 2010). “Bringing game theory into a course can help in two ways: by offering opportunities for active learning and helping students understand difficult concepts” (Ehrhardt, 2008, p. 60). However, the literature on PDG presents some criticalities. First, while game theory has a well-established place in the research literature (Axelrod and Hamilton, 1981; Sally, 1995), it still has not found a similar place in the literature about undergraduate training (Asal, 2005): there is still very little literature that considers its applications to education (Slater, 2004; Stull, 2006; Blake and Carroll, 2016).

Second, little space is dedicated to the use of PDG in an affective-emotional key. It is mainly treated with a cognitive key, to demonstrate that cooperation is the most convenient strategy (Dennis, 2015). It often focuses on individual characteristics (Hauert et al., 2006), building an internal model of the player's behavior that is at the same time coherent and compact (Gaudesi et al., 2014). Moreover, the goal of using PDG is to help students progress toward understanding a generalizable logic of social events (Morrow, 1994) or to study the level of cooperation and trust across different groups of people (Ahmed, 2008; Safin et al., 2013). However, instrumental rationality fails to explain intuitively obvious features of human interaction, thus requiring to incorporate nonstandard types of reasoning (Colman, 2003).

The psychoanalytic perspective, which emphasizes the emotional and irrational dimension of social interactions, uses PDG with a *descriptive* goal, which is far from the normative one, according to which the game “works” or is successful if participants are able to access cooperative logics. To this end, the game involves two groups, in which each one's success also depends on the choices of the other one. The iterative version of the game highlights the interdependence of social interactions: every choice of one group communicates to the other how it represents the relationship, even in absence of direct verbal communication. In repeated relationships where there is interdependence, representing others as an enemy does not allow participants to get satisfactory results.

In the psychoanalytic view, the game allows participants to experience the failure resulting from reading social functioning with primitive organizational skills, i.e. the primitive collusive models of representing others as an enemy (Carli and Giovagnoli, 2011).

Thus, the success of PDG does not coincide with the activation of cooperative modalities, but with the possibility to give voice to the sense of impotence evoked by the collusive failure. For this reason, it is necessary to pay attention and time to debriefing and class discussion, to foster the possibility of connecting theory to emotions and group decisions. According to Crookall, “the real learning comes not from the game, but from the debriefing, …[which] is the processing of the game experience to turn it into learning […] Debriefing is longer and more engaging for participants than the game itself” (2010, 907–908).

In light of the limitations present in the literature, the present study aims to explore:

- the significant characteristics of PDG used in an affective-emotional key in higher education;
- the learning outcomes that PDG enables to activate in the participants in relation to the development of organizational skills for conflict management.

This qualitative study could be considered a deepening of the results of our previous case study, that had supported the hypothesis that PDG has a facilitating effect on the quality of learning (Bruno and Dell'Aversana, 2018a).

## Method

### Participants and context

The context is a course of Organizational Psychology within a Master's Degree in the North of Italy, in the academic year 2014/15. Twenty-four students attended the course, mostly (80%) females, all with a bachelor's degree, 13 of them in Psychology, 11 in Social Work. The course lasted 10 weeks (18 meetings, 3 h each). In week 4, one meeting was devoted to PDG, to enhance organizational competences for conflict management.

PDG was proposed by dividing the class into 2 random groups, in order to present participants with identical intragroup and intergroup social dilemmas (Bornstein, 2003), with the following instruction for each group: “Your goal is to make the most points and lose the least possible; this, regardless of how much the other group gains or loses.”

The groups played 11 rounds and, after the eighth, they could choose to use a spokesperson to communicate directly with the other group. The last 3 rounds saw the points doubled and exhibited sharper “endgame” effects (Mason et al., 2014).

During the course, students were invited to write every week an e-journal without any spatial limitation about their learning experience, following each week a specific instruction. The requirement for the fourth week and to the week 9 was to describe the learning outcomes of the week. Journal entries of week 4 were considered for the analysis. Since journaling was not compulsory, 2 students did not write unit 4, and 2 students did not participate in the PDG meeting. This resulted in a corpus of 20 journal entries for analysis.

Following the completion of the course, all students were asked for written consent to have their journal entries included in this study.

### Thematic analysis

Journal entries were analyzed to gain comprehension of students' experience in relation to PDG by using N-Vivo Version 11. Thematic Analysis was performed to afford direct representation of individual point of view and description of experiences, beliefs and perceptions. A data-led approach was followed to maximize discovery and exploration (Braun and Clarke, 2006). Initially, journal entries were read completely to build the sense of experience, thoughts and emotions related to the training meeting. The relevant material was selected for each entry. This stage involved open coding. Coding was inductive, and represented recurrent and salient issues within the text, identified also by using word frequency query. After analyzing each journal entry, the codes were compared, and similar ones were grouped into broader categories. The identified themes captured important aspects of the data in relation to the research questions. Rival configurations of themes were discussed between the researchers and ultimately modified through agreement (Patton, 2002).

### Findings

Data analysis shows that PDG is perceived as a useful device in students' learning experience.

This kind of lessons, more interactive, stimulates me to reflect by researching in my personal experience situations in which I have had the opportunity to experience these concepts without even realizing it (6, f)

I really liked that this week ended like this (9, f)

Two main themes emerged from data analysis: (a) The significant dimensions of PDG experience; (b) New meanings about conflict.

### PDG significant dimensions: concreteness, intensity, debriefing

*Concreteness* refers to the learning of theoretical concepts otherwise difficult to use, due to their high level of abstraction.

The game placed us within an action perspective, without which I feel that I would not have fully understood the theory (5, f)

I could concretely test the meaning of the term collusion (10, f)

It also encourages the development of professional identity, thanks to the possibility of linking the experience in the classroom with experiences in social and organizational contexts.

This made us understand how a game can also be applied to everyday reality (15, f)

It made me reflect on how this process of simplification of reality also occurs within various organizational contexts. Starting from the simplest, like the university, in which often the goal changes becoming a challenge between comrades to get higher grades (7, f)

The second dimension, closely connected to the first, is *intensity*.

It is one of the first times that I get these aspects so clearly. The game in the classroom made me think a lot (3, f)

Today's game has shaken me (2, f)

Specifically, it refers to PDG capacity to recreate the emotional conditions of the conflict, with a particular emphasis on negative emotions, such as disappointment and shame.

Although it was just an exercise, the feeling that accompanied me after the end of the game was a strong feeling of regret because my group had not kept faith with the pact (3, f)

*On the basis of what emerged later in the plenary, I am a little ashamed to admit that initially I was an advocate not to send a representative to interview with the other because (throughout the game) I was convinced that the other team would have cheated (2, f)*.

Finally, *debriefing* emerged as essential to produce learning outcomes.

It was easy to understand, in retrospect, that collusive mode is easier than change (2, f)

*The most interesting part was the plenary session where aspects of the game emerged that during its development I could not understand (6, f)*.

### New meanings about conflict

PDG in higher education seems to sustain learning outcomes in three conceptual areas: collusion; competition; communication.

#### Collusion as unconscious modality of symbolizing the context

PDG facilitates the understanding of the concept of collusion. The students emphasize that it helped them to understand and experience unaware dynamics, their strength, automatism and the difficulty of modifying them, even if unsuccessful.

I thought I was a prisoner of my collusive process (5, f)

*Having lost sight of the instruction really surprised me because during the game I didn't realize it! (2, f)*.

#### Competition as automatism

PDG allowed students to experience that social phenomena are the effect not of single choices, but of social interactions, which are also driven by objectives that are not always explicit or conscious. Indeed, students realized that the competitive dimension arose automatically replacing the explicit goal of the game.

*My group at the beginning let itself be taken by the desire for victory, always aiming to make the other group succumb (1, f)*.

*As a consequence, we got caught by grudge (2, f)*.

In addition to their personal experience, students recognized several situations of social and organizational conflict.

The game made me understand what happens in everyday life: racism. To say that migrants “steal” the work of the Italians is the transformation of a nonzero-sum game into a zero-sum one. In fact, it is the mechanism of splitting, of collusion as a primitive and defensive function, which shows the other as an enemy (1, f)

*This week I have been able to reflect on how often, in organizations and groups, there are ways of symbolizing affectively the reality that become maladaptive (10, f)*.

#### Communication as organizational action

Furthermore, students refer to the attempt to overcome the competitive viewpoint, by evoking the opportunity to communicate between groups.

This activity was useful to understand that in the organizational action the choice of the other is important and represents a possible communication tool (9, f)

*The opportunity to meet the spokesman was not immediately welcomed by us, so I felt like insisting, to remind my team that this was an opportunity that was given to us (3, f)*.

## Discussion

PDG engages students in multidimensional aspects, which are cognitive, relational and affective, both on personal and professional dimensions. PDG seems to offer several interrelated advantages. On the methodological side, it seems to stimulate students' learning, due to different dimensions. The first one is “concreteness,” according to literature that recognizes that participatory game theory activities allow students to experience abstract concepts directly, giving them a reference to use when listening to explanations (Boyer et al., 2000; Tsekleves et al., 2016). The second one is “intensity” in line with recent research that recognizes the value of challenging situations for deep learning (Bjork and Linn, 2006). In contrast to other training tools, PDG provides a long-term perspective and experience of conflict in all its phases in a limited amount of time. The third one is that game design should start with the place where the participants are going to learn, that is, with debriefing (Crookall, 2010). It is the intersection between practice, emotion and reflection that allows students to build meaningful learning outcomes connected to their experience and future professional practice, as experiential learning approach has already shown (Bruno and Dell'Aversana, 2017, 2018b; Ripamonti et al., 2018). Indeed, debriefing allows to suspend the emotional collusion, to be able to think about it.

In relation to higher education outcomes, PDG seems to allow counterintuitive learning outcomes that are crucial in developing organizational competences for conflict management. First, it permits to elicit the individualistic stereotype that is used to analyze conflicts, especially by the classical organizational approach. On the contrary, PDG allows to highlight that social events are the epiphenomenon of interaction interdependence (Macy, 1991) and, more specifically, to experience one key dimension of social interdependence, that is its processual dimension. Second, the reference to the psychoanalytic approach to organizational studies permits to name the experience of using collusive modalities for emotionally symbolizing the context. It allows participants to feel that representing the other as a subject with which is possible to enter into the logic of exchange is the only chance to defend oneself from primitive “friend-enemy” relational modalities. Third, PDG highlights the importance of sustaining and protecting communication devices in organizational practices (Bruno and Bracco, 2016; Bruno et al., 2017), in line with experimental evidence that communication makes cooperation easier even without binding promises (Ostrom et al., 1992).

For these several reasons, PDG is useful for students' professional development to enlarge their organizational competences for conflict management. This issue seems to be relevant, since research reports that managers devote approximately one fifth of their time to handling conflict (Baron, 1989).

## Conclusion and limitation

In higher education, PDG allows to experience the challenge and the effort to curb and overcome the sense of powerlessness that organizational life continually activates in conflict management. Since many social situations are interpreted as zero-sum games instead of as nonzero-sum games (Carli, 2013), the reference to the psychoanalytic approach to organizational studies and, more specifically, to its concept of collusion as a way of symbolizing the context, is an answer to the urgent need of developing organizational competences for conflict management.

The study is a small-scale study and its findings cannot be generalized. However, it contributes to breaking PDG isolation in higher education literature (Asal, 2005; Ehrhardt, 2008; Blake and Carroll, 2016).

## Ethics statement

The study included a statement about personal data treatment, in accordance with the Italian privacy law (Law Decree DL-196/2003). Participants authorized the use of anonymous/collective data for scientific publications. Because the data were collected anonymously and the research investigated psychosocial concepts not adopting a medical perspective, ethical approval was not sought.

## Author contributions

AB and GD: conceptualized the study and chose the theoretical framework; GD and GG: analyzed the data; AB: supervised interpretation of data; AB: wrote the manuscript; AB, GD, and GG: improved and revised the manuscript.

### Conflict of interest statement

The authors declare that the research was conducted in the absence of any commercial or financial relationships that could be construed as a potential conflict of interest.
